# SWATH-MS-based quantitative proteomics reveals a uniquely intricate defense response in *Cnaphalocrocis medinalis*-resistant rice

**DOI:** 10.1038/s41598-020-63470-1

**Published:** 2020-08-05

**Authors:** Boon Huat Cheah, Hou-Ho Lin, Han-Ju Chien, Chung-Ta Liao, Li-Yu D Liu, Chien-Chen Lai, Ya-Fen Lin, Wen-Po Chuang

**Affiliations:** 1 Department of Agronomy, National Taiwan University, Taipei, 10617 Taiwan; 2 Institute of Molecular Biology, National Chung Hsing University, Taichung, 402 Taiwan; 3 Crop Enviroment Division, Taichung District Agricultural Research and Extension Station, Changhua County, 51544 Taiwan

**Keywords:** Plant molecular biology, Biotic

## Abstract

*Cnaphalocrocis medinalis* is a major insect pest of rice in Asia. A few defensive enzymes were reported to show higher activities in a resistant rice line (Qingliu) than in a susceptible rice line (TN1) upon leaffolder infestation. However, the overall molecular regulation of the rice defense response against leaffolder herbivory is unknown. Here, differential proteomic analysis by SWATH-MS was performed to identify differentially expressed proteins between the two rice varieties, Qingliu and TN1, at four time points of leaffolder herbivory, 0, 6, 24, and 72 h. Gene Ontology (GO) enrichment of the differentially expressed proteins indicated overrepresentation of (1) photosynthesis, (2) amino acid and derivative metabolic process, and (3) secondary metabolic process. Phenylalanine ammonia lyase and chalcone synthase, which catalyze flavonoid biosynthesis, and lipoxygenase, which catalyzes jasmonic acid biosynthesis, exhibited higher expression in Qingliu than in TN1 even before insect herbivory. Momentary activation of the light reaction and Calvin cycle was detected in Qingliu at 6 h and 24 h of insect herbivory, respectively. At 72 h of insect herbivory, amino acid biosynthesis and glutathione-mediated antioxidation were activated in Qingliu. A defense response involving jasmonic acid signaling, carbon remobilization, and the production of flavonoids and glutathione could underlie the resistance of Qingliu to leaffolder.

## Introduction

Rice production in Asia is affected by a harmful insect pest, *Cnaphalocrocis medinalis* (Guenée) (Lepidoptera: Pyralidae), also known as the rice leaffolder^1^. Leaffolder caterpillars feed on mesophyll tissues, interfering with photosynthesis and then reducing yield^2^. The affected leaf blades appear white, so heavily infested fields may have ‘scorched’-looking patches. This insect herbivore can complete three life cycles during each rice cropping season in Taiwan^3,4^. The application of chemical insecticides is the main method used to control pest populations in rice fields^5^. However, this method of pest control is rendered less effective because the caterpillars build a feeding chamber by folding a leaf longitudinally with silk, and the chamber indirectly protects the pests from the chemical spray.

When plants are infested by insect pests, they can differentiate diverse types of insects based on the insect elicitors or the nature of the damage caused by the insects^6^. The plants immediately activate various defensive signaling pathways, including those associated with mitogen-activated protein kinase (MAPK) and phytohormones, such as jasmonic acid (JA), salicylic acid (SA) and ethylene (ET)^7^. Chewing insects are generally known to trigger JA signaling pathways, while phloem-feeding insects trigger the SA signaling pathway^8^. The JA and SA signaling pathways also participate in antagonistic crosstalk in plant-insect herbivory interactions^8^. Because the signaling pathways triggered by different herbivorous insects vary, the production of downstream defensive proteins and secondary metabolites is unique to each insect attack.

Amino acid biosynthesis is upregulated by insect herbivory because amino acids serve as precursors for defensive plant metabolites^9,10^. Other primary metabolic pathways in plants, such as photosynthesis and carbon remobilization, are also reprogrammed during biotic stress^11^. For example, the expression of genes related to glycolysis, photosynthesis, carbohydrates and lipid metabolism in Arabidopsis is different between insect attacks by chewing herbivores and piercing-sucking aphids^12^. The biosynthesis of secondary metabolites is also induced when plants are exposed to insect infestation^13^. Phenolic compounds confer plant resistance against herbivores^14^. For example, lignin can increase leaf toughness, making the leaves more difficult for herbivores to chew^15^. The expression of genes encoding cinnamyl alcohol dehydrogenase (CAD), a lignin biosynthetic enzyme, was induced following herbivore-mediated damage in leaves^16^. Another two enzymes, polyphenol oxidase (PPO) and peroxidase (POD), catalyze the oxidation of phenols to form quinones^14^. Quinones bind covalently to leaf proteins and disturb protein digestion in insects^17^. Furthermore, flavonoids are a large family of polyphenolic plant compounds, and phenylalanine ammonia lyase (PAL), chalcone synthase (CHS) and chalcone-flavonone isomerase (CHI) are the key biosynthetic enzymes of flavonoids^18^. During insect herbivory, flavonoids are released by plants as either deterrents to pest oviposition and feeding or attractants to the natural predators of the pest^19^.

A recent study differentiated *C. medinalis*-resistant and *C. medinalis*-susceptible rice genotypes based on larval performance parameters, such as the percentage of rolled leaves^20^. The Qingliu (*indica*) rice genotype exhibited the lowest percentage of rolled-leaf damage inflicted by *C. medinalis*, indicating the resistance of this genotype to the pest. In our previous study, Qingliu (*C. medinalis*-resistant) showed a lower SPAD value, higher trichome density and higher defensive enzymatic activities of PPO, POD and PAL than Taichung Native 1 (TN1; *indica*; *C. medinalis*-susceptible)^21^. Moreover, our recent study reported a higher mortality rate and lower relative growth rate for caterpillars fed on Qingliu than for those fed on TN1^4^. Higher levels of PAL activity, jasmonyl-isoleucine and SA were also discovered in Qingliu compared to those in TN1 upon exposure to herbivory. A research gap still exists, as a comprehensive illustration of the complex regulatory network of the rice defense response against *C. medinalis* is not available.

In this study, we aimed to detect differentially expressed proteins between Qingliu and TN1 rice leaves upon exposure to herbivory by *C. medinalis* caterpillars at four different time points. SWATH-MS, a high-throughput and highly reproducible proteomic technology^22^, was used for this comparative study. This is the first proteomic profiling study targeted at elucidating the complex defense response of rice against *C. medinalis* herbivory.

## Results and Discussion

### An overview of the differentially expressed proteins between Qingliu and TN1 infested with *C. medinalis* caterpillars

A total of 1,732 quantified proteins were identified among the 24 duplicate samples (2 rice varieties, 4 time points, and 3 technical replicates) by carrying out spectral analysis and data interrogation with PeakView^®^ v.2.1 software. By shortlisting the quantified proteins with CV < 20% among the 24 duplicate samples as an assessment of assay reproducibility, a total of 633 quantified proteins was retrieved. After removing the unknown proteins annotated by GO, 505 proteins were retained for further analysis (Supplementary Table S1).

**Figure 1:**
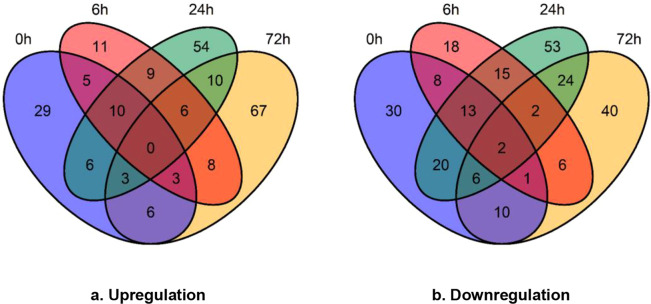
The numerical distribution of differentially expressed proteins in Qingliu compared with TN1 at the four time points of insect herbivory. (**a**) Upregulated and (**b**) downregulated proteins in Qingliu compared with TN1. A protein was considered differentially expressed if the fold change was >1.5 (for upregulation) or <0.67 (for downregulation), p-value <0.05 and CV < 20%. The blue, red, green and yellow sections of the Venn diagram represent the insect herbivory time points of 0, 6, 24 and 72 h, respectively.

**Figure 2:**
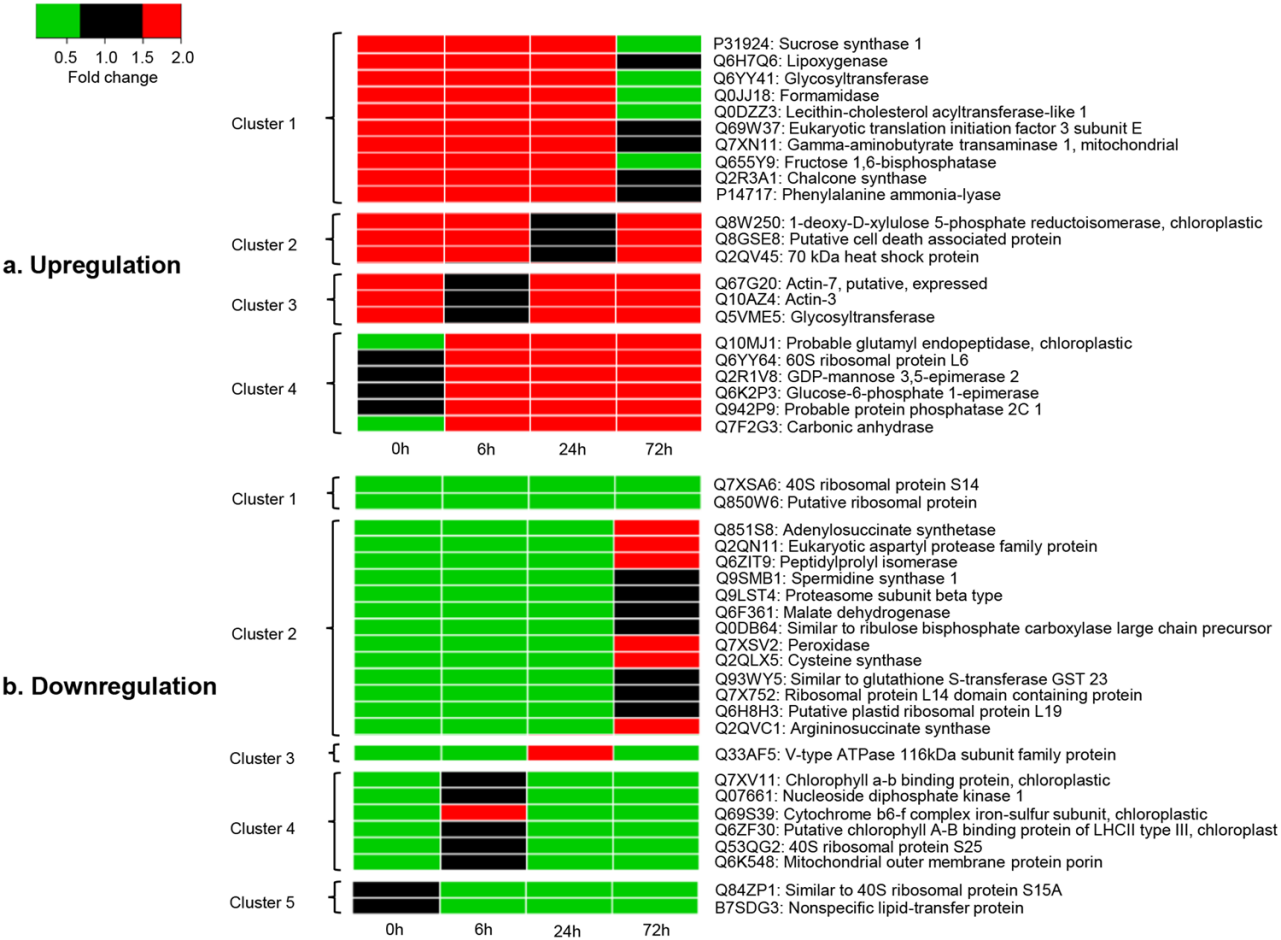
The heat map clustering of differentially expressed proteins in Qingliu compared with TN1 for at least three time points of insect herbivory. (**a**) Upregulated and (**b**) downregulated proteins in Qingliu compared with TN1. Red cells denote upregulation with fold change >1.5, black cells denote nondifferentially expressed (0.67 ≤fold change ≤1.5) and green boxes denote downregulation with fold change <0.67. Heat map clusters are shown on the left of heat maps, while UniProt ID and protein description are shown on the right of heat maps. The insect herbivory time points of 0, 6, 24 and 72 h represent the columns of the heat maps.

A total of 62, 52, 98 and 103 proteins displayed higher expression in Qingliu than in TN1 after 0, 6, 24 and 72 h of *C. medinalis* herbivory, respectively (Fig. 1a, Supplementary Table S2). Over 22 proteins were expressed at higher levels in Qingliu at three different time points and were categorized into four clusters (Fig. 2a). Cluster 1 represents upregulation at 0, 6, and 24 h; cluster 2 represents upregulation at 0, 6 and 72 h; cluster 3 represents upregulation at 0, 24 and 72 h; and cluster 4 represents upregulation at 6, 24 and 72 h. According to the functional information in UniProt, six proteins were related to carbon/sugar metabolism, viz., P31924, Q6YY41, Q655Y9, Q5VME5, Q6K2P3 and Q7F2G3 (Fig. 2a). In particular, fructose 1,6-bisphosphatase (Q655Y9) was mapped to the KEGG pathway osa00710: carbon fixation in photosynthetic organisms (Supplementary Fig. S1a), and glucose-6-phosphate 1-epimerase (Q6K2P3) was mapped to osa00010: glycolysis/gluconeogenesis (Supplementary Fig. S1b). The catalytic action of both enzymes was found to converge at the biosynthesis of fructose 6-phosphate (Supplementary Fig. S1a,b). Fructose 6-phosphate is an intermediate molecule in the process of ribulose-1,5-bisphosphate regeneration for carbon fixation. This finding suggests that carbon metabolism pathways, including the Calvin cycle, are activated in Qingliu under *C. medinalis* herbivory.

A total of 90, 65, 135 and 91 proteins showed lower expression in Qingliu than in TN1 after 0, 6, 24 and 72 h of insect herbivory, respectively (Fig. 1b, Supplementary Table S2). Over 24 were downregulated in Qingliu at three or more time points of insect herbivory, and these proteins were divided into five clusters (Fig. 2b). Cluster 1 represents downregulation at 0, 6, 24 and 72 h; cluster 2 represents downregulation at 0, 6 and 24 h; cluster 3 represents downregulation at 0, 6 and 72 h; cluster 4 represents downregulation at 0, 24 and 72 h; and cluster 5 represents downregulation at 6, 24 and 72 h. Cluster 1 contains two ribosomal proteins (Q7XSA6 and Q850W6) that were expressed at lower levels in Qingliu than in TN1 at all four time points (Fig. 2b). Another four ribosomal proteins (Q7X752, Q6H8H3, Q53QG2, Q84ZP1) also showed lower expression in Qingliu than in TN1 at three time points, namely, 0 h, 6 h, and 24 h (Fig. 2b). This might suggest that Qingliu and TN1 employed different translational regulation mechanisms in their respective interactions with *C. medinalis* herbivory. Based on the functional information in UniProt, four proteins (Q851S8, Q6F361, Q2QLX5, Q2QVC1) were associated with amino acid biosynthesis and are highlighted in cluster 2 (Fig. 2b). The expression levels of these proteins were lower in Qingliu than in TN1 at 0, 6 and 24 h but were higher in Qingliu at 72 h of insect herbivory.

Regarding the experimental design of this pioneering proteomic study, the early defense responses of Qingliu and TN1 to leaffolder herbivory were profiled at 0, 6 and 24 h, while their late defense responses were profiled at 72 h. Although 0 h was included, better results could have been derived by generating proteomic profiles of Qingliu and TN1 from the groups with and without insect damage at time points of 6, 24 and 72 h. Therefore, this is an important consideration in the experimental design of future studies on this research topic.

### GO enrichment reveals biological processes interrelated with *C. medinalis* herbivory resistance

**Figure 3:**
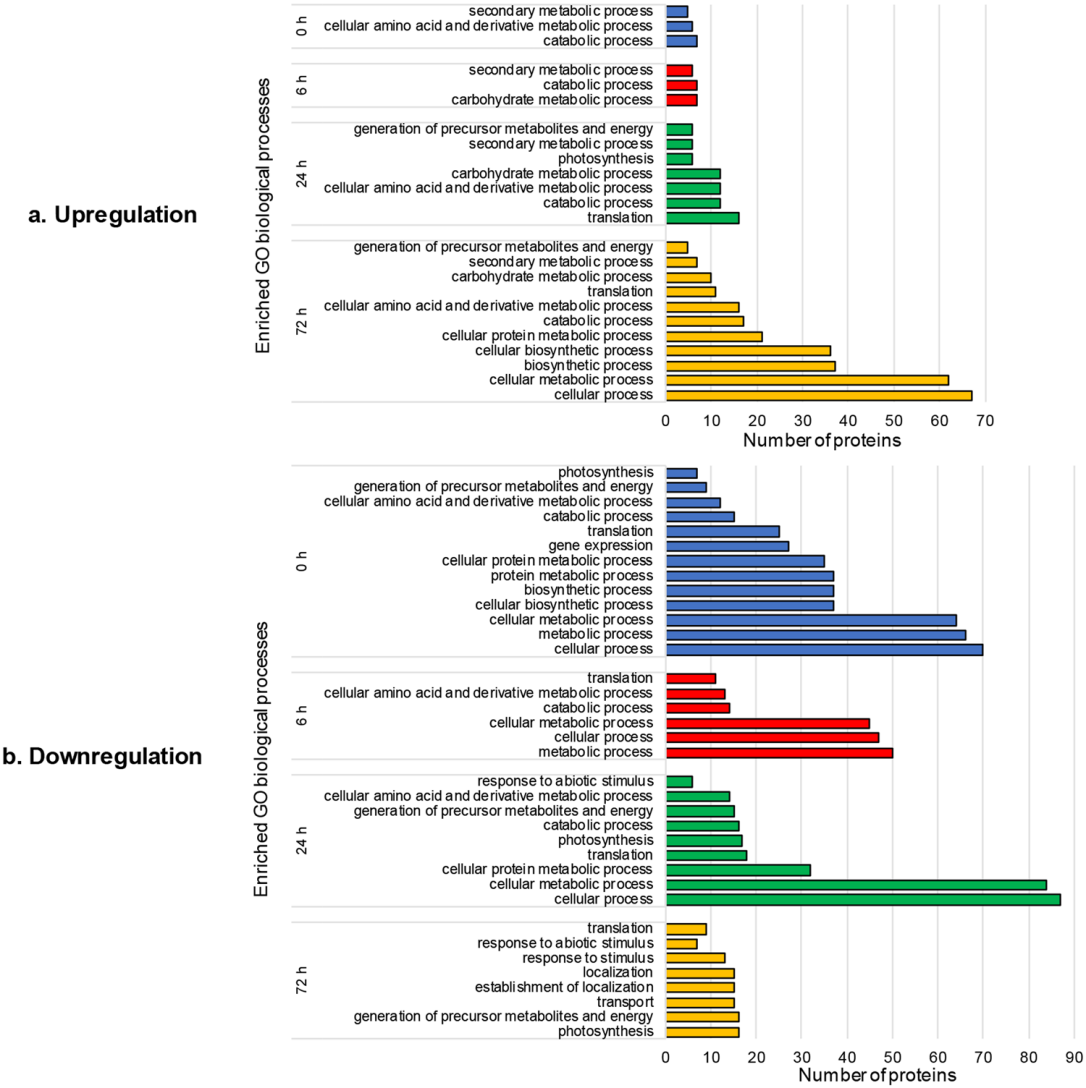
The number of differentially expressed proteins in Qingliu compared with TN1 that were annotated with enriched GO biological processes at each time point of insect herbivory (0, 6, 24 and 72 h). (**a**) Upregulated and (**b**) downregulated proteins in Qingliu compared with TN1. The GO enrichment analysis was performed through the Singular Enrichment Analysis (SEA) in agriGO v2.0 using Fisher’s Exact statistical test with Yekutieli (FDR under dependency) p-value <0.05.

Complete GO enrichment results of the differentially expressed proteins are shown in Supplementary Table S3. The enrichment of GO biological processes was emphasized (Fig. 3), as it provided insights into the differential mechanisms of *C. medinalis* resistance between Qingliu and TN1 (Supplementary Fig. S2).

GO biological process enrichment analysis was performed on the 62, 52, 98 and 103 proteins that were expressed at higher levels in Qingliu than in TN1 at 0, 6, 24 and 72 h of insect herbivory, respectively, and the results are shown in Fig. 3a (corresponding to Supplementary Fig. S2a,c,e,g for the respective time points). Overall, the numbers of annotated proteins and enriched GO biological processes increased gradually with the duration of insect herbivory. This result may indicate that Qingliu has the ability to respond to *C. medinalis* infestation by reprogramming its metabolic processes. Biological processes, such as secondary metabolic process, catabolic process, carbohydrate metabolic process, and cellular amino acid and derivative metabolic process, were enriched at multiple time points of insect herbivory (Fig. 3a); notably, secondary metabolic process and catabolic process were enriched at all four time points (Fig. 3a), i.e., 0 (Supplementary Fig. S2a), 6 (Supplementary Fig. S2c), 24 (Supplementary Fig. S2e) and 72 h (Supplementary Fig. S2g).

The separate GO enrichment (biological process) results for the 90, 65, 135 and 91 proteins with lower expression levels in Qingliu than in TN1 at 0, 6, 24 and 72 h of insect herbivory, respectively, are shown in Fig. 3b (corresponding to Supplementary Fig. S2b,d,f,h for the respective time points). Photosynthesis and cellular amino acid and derivative metabolic process were enriched for the downregulated proteins in Qingliu at multiple time points of insect herbivory (Fig. 3b). For instance, enrichment of photosynthesis was observed for the downregulated proteins in Qingliu at 0 (Supplementary Fig. S2b), 24 (Supplementary Fig. S2f) and 72 h (Supplementary Fig. S2h) of herbivory, whereas enrichment of this process was detected only at 24 h of herbivory for the upregulated proteins in Qingliu (Fig. 3a, Supplementary Fig. S2e). This result indicates that there are two sets of photosynthesis-related proteins that were differentially expressed between Qingliu and TN1 at 24 h of insect herbivory. The cellular amino acid and derivative metabolic process term was enriched for the downregulated proteins in Qingliu at 0, 6 and 24 h of insect herbivory (Fig. 3b, Supplementary Fig. S2b,d,f) and for the upregulated proteins in Qingliu at 0, 24 and 72 h of insect herbivory (Fig. 3a, Supplementary Fig. S2a,e,g). As amino acid metabolism is pertinent to plant defense against insect herbivory, this comparison reveals a complex set of associated differentially expressed proteins between Qingliu and TN1.

### Insight into the proteins that regulate the enriched defense-related biological processes

#### Photosynthesis

Plants undergo complex changes in photosynthesis and carbon remobilization during insect herbivore attacks because photoassimilates are important substrates for growth and energy storage^11^. Photosynthesis is postulated to be activated following insect herbivory for one of these reasons: (1) the synthesis of defensive metabolites requires organic compounds; (2) the reduced total surface area of leaves following insect infestation increases the rate of photosynthesis in the nondamaged leaves; and (3) insect herbivores can regulate photosynthesis as an exploitative strategy to maximize food resources^11^. Another theory postulates that photosynthesis is suppressed following insect herbivory because (1) plants have to conserve energy for the initiation of defense responses; (2) injured leaves undergo senescence, apoptosis and abscission; and (3) the suppression of photosynthesis can reduce food availability for insect pests^11^.

In this study, a total of 27 proteins with differential expression between Qingliu and TN1 at four time points of *C. medinalis* herbivory were annotated as being involved in photosynthesis (Supplementary Table S4a). Before insect herbivory (0 h), seven proteins were expressed at lower levels in Qingliu than in TN1, while two proteins were expressed at higher levels in Qingliu than in TN1 (Supplementary Table S4a). Intriguingly, at 6 h of insect herbivory, four proteins were upregulated in Qingliu compared with TN1 (Supplementary Table S4a). At 24 h, the expression of seventeen proteins was lower in Qingliu than in TN1, whereas the expression of six proteins was higher in Qingliu than in TN1 (Supplementary Table S4a). At 72 h, sixteen proteins exhibited lower expression in Qingliu than in TN1, while one protein was expressed at higher levels in Qingliu than in TN1 (Supplementary Table S4a). Although the differential expression profile of the 27 photosynthesis-associated proteins is complex, the proteins could be categorized into either light or dark reactions.

A literature search showed that among the 27 proteins annotated to photosynthesis in this study, over 18 proteins are affiliated with light reactions, and two proteins are affiliated with dark reactions (Supplementary Table S4a). Among the 53 observations of differential expression, only 13 observations (13/53*100 = 24.5%) showed higher protein expression in Qingliu than in TN1 (Supplementary Table S4a). For each time point, only 22.2%, 26.1% and 5.9% of the differentially expressed proteins were upregulated in Qingliu compared with TN1 at 0, 24 and 72 h, respectively (Supplementary Table S4a). Interestingly, the opposite trend was observed at 6 h of insect herbivory, wherein four proteins (Q7F4T1, P41344, P12330, Q69S39) were upregulated in Qingliu compared with TN1 (Supplementary Table S4a). The four upregulated proteins in Qingliu consist of the structural components of chlorophyll or the photosystem and enzymes that catalyze the electron transport chain (Supplementary Table S4a). This finding may indicate that the light reaction of photosynthesis was activated in Qingliu at 6 h of insect herbivory.

Subsequently, at 24 h of insect herbivory, 17 proteins were downregulated in Qingliu compared with TN1, while six proteins were upregulated in Qingliu compared with TN1 (Supplementary Table S4a). Notably, the two dark reaction-affiliated proteins (O64422, Q9ZTP5) were differentially expressed only at this time point (Supplementary Table S4a). Fructose-1,6-bisphosphatase (O64422) was upregulated in Qingliu compared with TN1, whereas ribulose-phosphate 3-epimerase (Q9ZTP5) was downregulated in Qingliu compared with TN1 (Supplementary Table S4a). Fructose-1,6-bisphosphatase catalyzes the conversion of fructose-1,6-bisphosphate to fructose 6-phosphate to form the CO_2_ acceptor molecule in the Calvin cycle, ribulose-1,5-biphosphate^23^. Therefore, the relatively high expression of this enzyme in Qingliu resulted in an elevated level of carbon fixation. Next, ribulose-phosphate 3-epimerase catalyzes the interconversion between ribulose 5-phosphate and xylulose 5-phosphate^24^. As ribulose 5-phosphate is an intermediate substrate in the Calvin cycle, the relatively low expression of ribulose-phosphate 3-epimerase in Qingliu might favor the accumulation of ribulose 5-phosphate. Collectively, these findings indicate that the Calvin cycle is activated in Qingliu at 24 h of insect herbivory. One of the beneficial outcomes of the activation of the Calvin cycle is induction of the biosynthesis of amino acids and other metabolites that might be crucial for the resistance of Qingliu to *C. medinalis* herbivory^25^.

#### Amino acid and derivative metabolic process

Amino acids play an important role in plant-insect interactions, both as a source of nutrients for growth and as precursors for plant defensive metabolites^9,10^. Therefore, when plants are attacked by herbivorous insects, one of the responses is the reprogramming of amino acid biosynthesis as a counteractive measure to restrict the supply of amino acids for insect pests^26^. Over 41 proteins differentially expressed between Qingliu and TN1 upon exposure to *C. medinalis* herbivory for the four time points were annotated to amino acid and derivative metabolic process (Supplementary Table S4b). Prior to insect feeding (0 h), six proteins were expressed at a higher level in Qingliu than in TN1, while twelve proteins were expressed at a lower level in Qingliu than in TN1 (Supplementary Table S4b). At 6 h of insect herbivory, four proteins were expressed at a higher level in Qingliu than in TN1, while twelve proteins were expressed at a lower level in Qingliu than in TN1 (Supplementary Table S4b). At 24 h, twelve proteins and fourteen proteins were up- and downregulated in Qingliu in comparison with TN1, respectively (Supplementary Table S4b). At 72 h of insect herbivory, sixteen proteins and two proteins were up- and downregulated in Qingliu in comparison with TN1 (Supplementary Table S4b).

In regard to amino acid biosynthesis, an interesting trend was observed, wherein a set of enzymes (Q65XK0, Q7XS58, P14654, Q5JNB0, Q6AV34, Q7F2F8) was expressed at lower levels in Qingliu than in TN1 at 0, 6 and/or 24 h of insect herbivory (Supplementary Table S4b). Conversely, another set of amino acid biosynthetic enzymes (Q69RJ0, Q10NY1, Q5VNW0, Q7Y096, Q6L4H5, Q10NW0, Q6KAJ2, Q2QLX5, Q2QVC1) was discovered to be induced in Qingliu in comparison with TN1 at either 24 or 72 h of insect herbivory (Supplementary Table S4b). For instance, the expression of cysteine synthase (Q2QLX5) was lower in Qingliu than in TN1 at 0, 6 and 24 h of insect herbivory (Supplementary Table S4b). However, the protein was expressed at higher levels in Qingliu than in TN1 at 72 h (Supplementary Table S4b). Similarly, other enzymes responsible for the biosynthesis of isoleucine (Q65XK0), cysteine (Q7XS58, Q5JNB0), glutamine (P14654), arginine (Q6AV34) and methionine (Q7F2F8) were expressed at lower levels in Qingliu than in TN1 at 0, 6 and/or 24 h of insect herbivory (Supplementary Table S4b). Furthermore, a mutually exclusive set of biosynthetic enzymes for glutamine (Q69RJ0), leucine (Q7Y096), threonine (Q6L4H5), histidine (Q10NW0), aspartate (Q6KAJ2) and argininosuccinate (Q2QVC1) as well as chorismate (Q10NY1, Q5VNW0), a precursor of aromatic amino acids such as phenylalanine, tryptophan and tyrosine (Supplementary Table S4b), were expressed at higher levels in Qingliu than in TN1 at either 24 or 72 h of insect herbivory. Based on this discovery, we postulate that Qingliu can, at the expense of its primary metabolism, temporarily repress its biosynthesis of amino acids to limit the supply of both essential and nonessential amino acids to *C. medinalis* at 0, 6 and 24 of insect herbivory. This mechanism could potentially jeopardize the normal growth of *C. medinalis* and hence weaken its feeding vigor. Then, at an opportune moment, i.e., at 24 and 72 h of *C. medinalis* herbivory, Qingliu induces amino acid biosynthesis through another set of biosynthetic enzymes to compensate for the loss of primary metabolism and produce defensive secondary metabolites.

Four glutathione S-transferases were detected in the dataset (Supplementary Table S4b), implying that glutathione may play a role in protecting the cellular components of rice plants from the damage caused by reactive oxygen species (ROS) during *C. medinalis* infestation (Supplementary Table S4c). Glutathione peroxidases can reduce H_2_O_2_ to water through the oxidation of reduced glutathione (GSH) to glutathione disulfide (GSSG)^27^. Glutathione reductase, in turn, reduces the oxidized GSSG back to GSH with the reducing power of NADPH^28^. GSH is a well-known tripeptide that is closely associated with plant defense against herbivorous insects^29^. For example, an Arabidopsis glutathione-deficient mutant, *pad2-1*, was found to accumulate glucosinolates at lower levels than its wild-type counterpart and hence was more susceptible to infestation with *Spodoptera littoralis*^30^. GSH can eliminate ROS and is positively regulated by methyl jasmonate^31^. Furthermore, the function of glutathione peroxidases is also relevant to plant-insect interactions^32^. Herbivory by grain aphids on maize seedlings induced the expression of genes encoding glutathione peroxidases, viz., ZmGPX1 and ZmGPX3. The activity of the two encoded enzymes was also increased, which in turn triggered the antioxidant response^33^. Glutathione S-transferase plays a fundamental role in the elimination of toxic compounds and ROS from plants by catalyzing the conjugation of these substances with GSH. Compared with uninfested Arabidopsis plants, Arabidopsis plants infested with diamondback moths (*Plutella xylostella*) exhibited higher expression of glutathione S-transferase^34^.

In this study, glutathione reductase (P48642, Q8S5T1) was not differentially expressed between Qingliu and TN1 under insect herbivory (Supplementary Table S4c). Glucose-6-phosphate 1-dehydrogenase (Q7X7I6) catalyzes the rate-limiting step in the pentose phosphate pathway, through which NADPH is provided as a reducing agent for GSSG^35^. At 6 and 24 h of insect herbivory, glucose-6-phosphate 1-dehydrogenase was expressed at higher levels in Qingliu than in TN1, with fold change values of 1.6 and 1.8, respectively (Supplementary Table S4c). This finding indicates that an adequate supply of NADPH is acquired by Qingliu as a reducing agent for the formation of the reduced GSH from GSSG. Four proteins that are related to glutathione S-transferase (Q93WY5, Q7XCK0, Q945W5, Q8H8D8) displayed a trend wherein their expression was generally lower in Qingliu than TN1 at 0, 6 and 24 h (Supplementary Table S4c) but higher in Qingliu than in TN1 at 72 h (Supplementary Table S4c). It can be deduced that the antioxidative function of glutathione S-transferase is required in Qingliu at the late stage of insect herbivory.

#### Secondary metabolic process

Plants produce various types of secondary metabolites that act as defensive compounds against herbivores and microbes^13^. Altogether, 11 differentially expressed proteins between Qingliu and TN1 following the four time points of *C. medinalis* herbivory were annotated to the secondary metabolic process category (Supplementary Table S4d). Prior to insect herbivory (0 h), the expression levels of five proteins were relatively high in Qingliu compared with TN1 (Supplementary Table S4d). At 6 h of insect herbivory, six proteins were upregulated in Qingliu compared with TN1, while two proteins were downregulated in Qingliu compared with TN1 (Supplementary Table S4d). At 24 h, six proteins were upregulated in Qingliu compared with TN1, while one protein was downregulated in Qingliu compared with TN1 (Supplementary Table S4d). At 72 h, seven proteins were upregulated in Qingliu compared with TN1, while one protein was downregulated in Qingliu compared with TN1 (Supplementary Table S4d). Notably, among the 28 observations of differential expression (by counting the total occurrence of red and green highlighted fold changes for the 11 differentially expressed proteins between Qingliu and TN1 (Supplementary Table S4d), 24 observations (24/28*100 = 85.7%) were derived from proteins that were upregulated in Qingliu compared with TN1 upon insect herbivory. A previous study reported that the expression of CAD, which catalyzes the final step in the synthesis of lignin monomers, was induced in leaves upon herbivorous attack^16^. Consistent with this study, a probable CAD3 (Q337Y2) was downregulated in Qingliu compared with TN1 at 6 h of insect herbivory but upregulated in Qingliu compared with TN1 at 72 h of insect herbivory.

**Figure 4:**
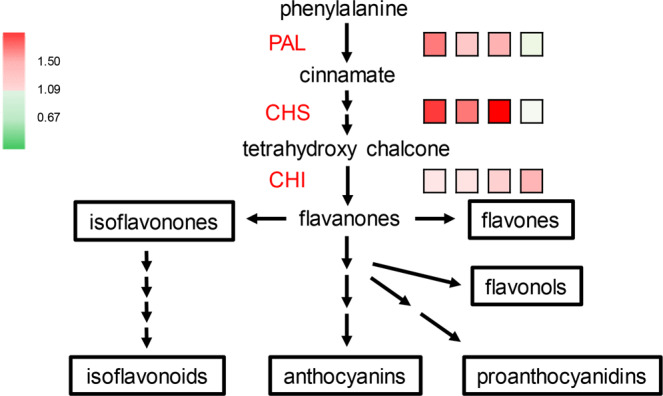
Flavonoid biosynthesis. The expression fold change in phenylalanine ammonia lyase (PAL), chalcone synthase (CHS) and chalcone-flavonone isomerase (CHI) between Qingliu and TN1 at 0, 6, 24, and 72 h of insect herbivory is shown by the square boxes from left to right accordingly. Red boxes denote upregulation with a 1.5-fold change, while green boxes denote downregulation with a 0.67-fold change.

Several major subclasses of flavonoids, namely, flavonols, flavones, proanthocyanidins, flavan-3-ols, flavonones, flavans and isoflavonoids, are associated with plant defense against insect feeding^14^. Our results showed that 3 out of 11 (3/11*100 = 27.3%) of the proteins were directly engaged in flavonoid biosynthesis (Fig. 4, Supplementary Table S4d). In the flavonoid biosynthetic pathway (Fig. 4), PAL catalyzes the committed step for the breakdown of phenylalanine to cinnamic acid^36^. Cinnamic acid is then converted to chalcone through catalysis by CHS^37^. Chalcone is in turn isomerized to flavonone through catalysis by CHI^38^. Flavonones are precursors of other flavonoid compounds, such as anthocyanin, isoflavonoids and flavones^39^. The expression of PAL (P14717) was higher in Qingliu than in TN1 at 0, 6 and 24 h of insect herbivory, with fold change values of 2.6, 1.7 and 2.0, respectively (Fig. 4, Supplementary Table S4d). Furthermore, CHS (Q2R3A1) was also upregulated in Qingliu compared with TN1 at 0, 6 and 24 h of insect herbivory (Fig. 4, Supplementary Table S4d). The highest fold change in CHS levels was 18.8 at 24 h, in comparison to 3.4 at 0 h and 2.6 at 6 h. PAL and CHS were not differentially expressed between the two rice varieties at 72 h of insect herbivory (Fig. 4, Supplementary Table S4d), mainly due to the subsequent increase in the expression of both proteins in TN1. Nevertheless, notably, the highest PAL expression in TN1 at 72 h was still lower than the highest PAL expression in Qingliu. It can be deduced from these findings that PAL and CHS are expressed at higher levels in the *C. medinalis*-resistant Qingliu than in TN1 even prior to insect herbivory.

The accumulation of these enzymes, PAL and CHS, can potentially enhance Qingliu resistance to leaf feeding by *C. medinalis* by priming the biosynthesis of essential flavonoids at the early stages of insect herbivory. Meanwhile, the rate of downstream flavonoid biosynthesis is determined by CHI due to its involvement in the catalysis of the rate-limiting step^40^. In this study, the expression of CHI (Q84T92) was also higher in Qingliu than in TN1 at 24 and 72 h of insect herbivory, with fold changes of 1.6 and 1.9, respectively (Fig. 4, Supplementary Table S4d). Taken together, the results show that the relatively high expression of PAL and CHS in Qingliu before (0 h) and after insect herbivory (6, 24 h) is corroborated by the eventual increase in the expression of CHI at 24 and 72 h. In short, these results showed not only that flavonoid biosynthesis is integral to Qingliu resistance against *C. medinalis* herbivory but also that the rate or timing at which this secondary metabolic pathway is activated could be the determining factor.

#### Jasmonic acid (JA) pathway

Under herbivory of chewing insects, the JA signaling pathway in plants is triggered to activate the production of defensive proteins that can impede the digestion and growth of insect pests^8,41^. In addition, our recent study demonstrated that Qingliu exposed to 6 h of herbivory by *C. medinalis* produced higher levels of jasmonyl-isoleucine than TN1^4^. Therefore, JA signaling is discussed here even though our GO enrichment analysis did not reveal overrepresentation of this pathway.

**Figure 5:**
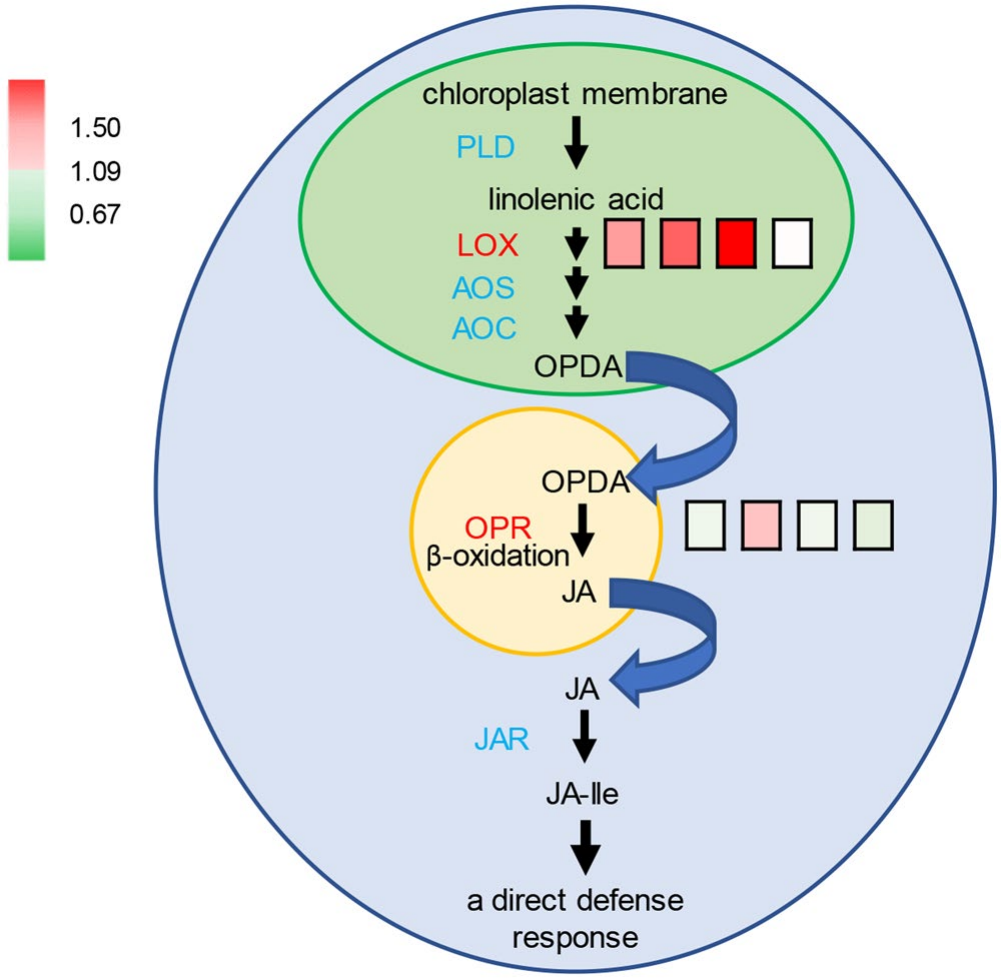
JA biosynthetic pathway and its involvement in orchestrating plant direct defense response. The expression fold change in LOX and OPR between Qingliu and TN1 at 0, 6, 24, and 72 h of insect herbivory is shown by square boxes from left to right accordingly. Red boxes denote upregulation with a 1.5-fold change, while green boxes denote downregulation with a 0.67-fold change. Chloroplasts, peroxisomes and cytoplasm are represented by green, orange and blue compartments, respectively. PLD = phospholipase, LOX = lipoxygenase, AOS = allene oxide synthase, AOC = allene oxide cyclase, OPDA = 12-oxo-phytodienoic acid, OPR = 12-oxo-phytodienoic acid reductase, JAR = acyl adenylate-forming enzyme, JA-Ile=conjugated jasmonyl-isoleucine.

A JA biosynthetic enzyme, lipoxygenase (LOX; Q6H7Q6), was expressed at higher levels in Qingliu than in TN1, with fold change values of 2.2, 2.9 and 15.7 at 0, 6 and 24 h of insect herbivory, respectively (Fig. 5, Supplementary Table S4e). LOX expression became comparable between Qingliu and TN1 at 72 h (Fig. 5, Supplementary Table S4e). Therefore, it can be inferred from this observation that LOX is expressed at a higher level in Qingliu at the uninfested (0 h) and early stages (6, 24 h) of insect herbivory. Another JA biosynthetic enzyme, OPDA reductase 1 (OPR1; Q84QK0), was expressed at higher levels in Qingliu than in TN1, with a fold change of 1.8 at 6 h of insect herbivory (Fig. 5, Supplementary Table S4e). Taken together, these findings suggest that Qingliu is capable of triggering its JA-mediated defense machinery against *C. medinalis* faster than TN1. This unique capability of Qingliu may explain the lowered relative growth rate (RGR) of insects observed in Qingliu compared with TN1 in our previous study^4^.

## Conclusion

This differential proteomic study of *C. medinalis*-resistant Qingliu and *C. medinalis*-susceptible TN1 at different time points of insect herbivory has provided us with a time course simulation of the mechanism by which important biological processes are differentially regulated between the two varieties at the protein level. The key enzymes involved in flavonoid (PAL and CHS) and JA (lipoxygenase) biosynthesis were expressed at higher levels in Qingliu than in TN1 even before insect herbivory (0 h). The upregulation of these enzymes in Qingliu could also be detected at 6 and 24 h of insect herbivory. This unique expression profile in Qingliu might prime the activation of flavonoid biosynthesis and JA signaling and thus account for the resistance of Qingliu to insect herbivory. Momentary activation of the light reaction and momentary activation of the Calvin cycle were detected in Qingliu at 6 h and 24 h, respectively. Subsequently, at 72 h of insect herbivory, the activation of amino acid biosynthesis and glutathione-mediated antioxidation occurred in Qingliu.

## Methods

### Plant materials

Two rice (*O. sativa* L.) varieties, namely, Qingliu and TN1, were used for this study. Qingliu is a *C. medinalis***-**resistant variety^20^, whereas TN1 is a *C. medinalis***-**susceptible variety^21^. Seeds were first surface sterilized with 2% (v/v) NaOCl for 40 min and further washed in distilled water. The sterilized seeds were germinated on water**-**moistened paper towels in Petri dishes under dark conditions at 37 °C for 48 h. After germination, seeds with uniform size were transferred to 1 l polyethylene pots containing 700 ml of sterile vermiculite soaked with 1X Kimura B nutrient solution [(NH_4_)_2_SO_4_, 365 µM; KNO_3_, 183 µM; MgSO_4_.7H_2_O, 548 µM; KH_2_PO_4_, 182 µM; Fe-citrate, 61.2 µM; Ca(NO_3_)_2_.4H_2_O, 365 µM; H_3_BO_3_, 2.51 µM; MnSO_4_.H_2_O, 0.20 µM; ZnSO_4_.7H_2_O, 0.20 µM; CuSO_4_.5H_2_O, 0.05 µM; H_2_MoO_4_, 0.05 µM]^42^. Each 1 l pot (64 mm diameter at the base, 95 mm diameter at the aperture, and 165 mm height) contained two seedlings. The 1X Kimura B nutrient solution was changed every two days. Plants were grown in a growth chamber set at 30/25 °C (day/night) with a 12/12 h (day/night) photoperiod and a light intensity of 220 μmol m^−2^s^−1^. Plants were used for experiments 30 days after germination.

### Insect management

A *C. medinalis* colony was obtained from Taichung District Agricultural Research and Extension Station, COA, Changhua, Taiwan, and maintained on maize seedlings (White Pearl, Known**-**You Seed Co., Taiwan) by following a modified maize seedling rearing method^43^ in a growth chamber set at 30/25 °C (day/night) with 55 ± 5% relative humidity and a 12/12 h (day/night) photoperiod. Moths were fed with a 10% (v/v) sucrose solution.

Third-instar *C. medinalis* caterpillars were used in this study. Caterpillars were starved for 4 h before being placed, one caterpillar per plant, on the newly expanded leaves of 30**-**day**-**old rice plants (at the six**-**leaf stage). A plastic cover with mesh cloth was used to cover each pot to prevent caterpillars from escaping. For both varieties, the infested area of the newly expanded leaf was excised and collected from each infested rice plant at 6, 24 and 72 h after infestation. For 0 h infestation controls, the newly expanded leaf of each uninfested plant was excised at the location that corresponds to the infested area of a treated plant. The early plant defense responses to leaffolder herbivory were profiled at 0, 6 and 24 h, while the late defense responses were profiled at 72 h. For each variety, six excised leave samples (each from an individual plant) from 0 h were pooled, whereas eight excised leave samples (each from an individual plant) from 6, 24 and 72 h were pooled accordingly. The pooled samples were immediately frozen in liquid nitrogen and stored at −80 °C before analysis.

### Protein extraction

The protein extraction method was modified according to the protocol described in Liu *et al*.^44^. Each pooled sample (approximately 0.5 g) was ground in liquid nitrogen using a 2010 Geno/Grinder (SPEX SamplePrep, NJ, USA). The homogenates were mixed with 21 ml of leaf extraction buffer (0.7 M sucrose, 0.5 M Tris**-**base, 50 mM EDTA, 0.1 M KCl, 30 mM HCl, 2% β**-**mercaptoethanol), 0.5 g of polyvinylpolypyrrolidone (PVPP), and 250 µl of 40 mM phenylmethanesulfonyl fluoride (PMSF) and then centrifuged at 12,000 rpm for 20 min at 4 °C. The supernatant was transferred to a new centrifuge tube before mixing with 16 ml of water**-**saturated phenol at 4 °C for 5 min and then centrifuged at 12,000 rpm for 20 min at 4 °C. After being transferred to a new centrifuge tube, the supernatant was mixed with 16 ml of leaf extraction buffer at 4 °C for 5 min before centrifugation at 12,000 rpm for 20 min at 4 °C. The mixing of the supernatant with leaf extraction buffer was repeated with 10 ml of leaf extraction buffer. The supernatant was transferred to a new centrifuge tube and then mixed with 30 ml of 0.1 M NH_4_OAc/methanol/10 mM β**-**mercaptoethanol. The mixture was kept at **-**20 °C for 16 h to allow precipitation to occur and then centrifuged at 12,000 rpm for 20 min at 4 °C. The supernatant was discarded, and the pellet was dissolved in 1.5 ml of 0.1 M NH_4_OAc/methanol/10 mM β**-**mercaptoethanol. The mixture was transferred to a new centrifuge tube and centrifuged at 8,000 rpm for 5 min at room temperature. The supernatant was discarded, and the pellet was mixed with 1 ml of 100% acetone/10 mM β**-**mercaptoethanol before centrifugation at 8,000 rpm for 5 min at room temperature. This washing process was repeated three times before the mixture was vacuum dried. The dried powder was dissolved in 40 µl of lysis buffer (9.5 M urea, 4% NP**-**40, 4% Triton X**-**100, 6 mM dithiothreitol, 40 mM Tris**-**base). The protein concentration was quantified using the Bradford method^45^. The quantified protein samples were mixed with protease inhibitor cocktail and 40 mM PMSF, then stored at **-**80 °C until further analysis.

### SDS-PAGE analysis and in-gel tryptic digestion

The proteins were concentrated by 28% SDS**-**PAGE and then visualized by silver staining. The candidate bands were excised carefully and collected in tubes for digestion. Proteins were washed three times with 500 μl of ddH_2_O, with each wash lasting for 30 min. The ddH_2_O was discarded, and the proteins were washed three times with 200 μl of 50 mM ammonium bicarbonate (ABC), with each wash lasting for 5 min. The 50 mM ABC was then discarded, and the proteins were further washed with 200 μl of 100 mM ABC/50% acetonitrile (ACN) for 5 min and dehydrated with 100% ACN until they turned white. The proteins were reduced with 50 mM dithiothreitol (DTT) and then alkylated with 100 mM iodoacetamide. The digestion solution (50 mM ABC) containing trypsin (2 ng/μl) was added to the tubes, and the tubes were incubated with shaking at 37 °C for 16 h. The extracted digests were vacuum dried. After digestion, the tryptic peptides were prepared by suspending the extracts with 0.1% formic acid (FA) or were stored at **-**20 °C until nano-LC**-**MS/MS analysis.

### LC-MS/MS analysis

Peptides were dissolved in 0.1% FA, and 250 fmole of digested beta**-**galactosidase was added to each 1 μg of protein sample as an internal standard for retention time (RT) and intensity calibration. Peptides resulting from the digestion of the protein mixture were analyzed by using a trap column (C18, 100 μm x 2 cm nanoViper, Thermo Fisher Scientific, MA, USA) and an analytical column (C18, 75 μm x 25 cm nanoViper, Thermo Fisher Scientific, MA, USA). A binary solvent system consisting of 0.1% FA in ddH_2_O and 0.1% FA in ACN (mobile phase B) was used to separate peptides through the nano**-**LC system. A flow rate of 300 nl/min on a Thermo Scientific Dionex UltiMate^TM^ 3000 RSLCnano system was used with the following gradient: 5% mobile phase B for 3.5 min, followed by a linear gradient of 5**–**30% mobile phase B within 170 min, a second linear increase to 60% mobile phase B within 5 min, a third linear increase to 90% mobile phase B within 1 min, a hold at 90% mobile phase B for 20 min, a fourth linear decrease to 5% mobile phase B within 1 min, and then a hold at 5% mobile phase B for 13 min. The total LC time was 208 min. Eluents from the column were analyzed by a TripleTOF 6600 system (SCIEX) in positive ion mode with a nano**-**ion spray voltage of 2800 V. Data-dependent acquisition (DDA) was performed to generate the SWATH spectral library. A full scan of 250 ms (TOF**-**MS) in the range of 350**–**1250 m/z was collected from the MS1 spectra. The top 30 precursor ions with charge states from +2 to +4 were selected for fragmentation with an accumulation time of 80 ms per MS/MS experiment for a total cycle time of 2.65 s. Then, the exclusion time of 25 s was acquired in the range 65**–**1800 m/z. The SWATH analysis parameters were as follows: full scan of 40 ms (TOF**-**MS) in the range of 350**–**1250 m/z and all precursor ion fragmentation with an accumulation time of 80 ms. The 25 Da windows spanned the mass range 350**–**1250 m/z. The SWATH and DDA procedures were performed in high-sensitivity mode. All DDA data files were used by ProteinPilot^TM^ (SCIEX) to generate a reference spectral library. Subsequently, the DIA data and spectral library were loaded into PeakView^®^ v.2.1 software with SWATH^TM^ acquisition v.2.0 (SCIEX) under stringent criteria and settings: six peptides, six transitions, 95% peptide confidence, and an ion library mass tolerance of 0.01 Da. All shared peptides were excluded from the analysis. The output of quantified protein areas was normalized with MarkerView^TM^ v.1.3 (SCIEX) by total area sum normalization. Each of the eight biological samples (2 rice varieties and 4 time points) was analyzed with SWATH-MS in three technical replicates. Proteins with a fold change of> 1.5 or <0.67 (p**-**value <0.05, coefficient of variation (CV) < 20%) were considered differentially expressed proteins in this research. The differentially expressed proteins for each time point of insect herbivory were classified with a Venn diagram (http://bioinformatics.psb.ugent.be/webtools/Venn/). Heat maps of the fold change in expression were generated using the heatmap.2 function of the gplots package in R^46^.

### Gene Ontology (GO) enrichment and functional analyses

A list containing the Universal Protein Resource (UniProt) accessions of differentially expressed proteins (up**-** or downregulated) between Qingliu and TN1 was prepared for each time point of insect herbivory (0, 6, 24, 72 h). Therefore, a total of eight lists were used separately as input files for Gene Ontology (GO) enrichment analysis, which was performed through Singular Enrichment Analysis (SEA) in agriGO v2.0^47^. The parameters used were as follows: (1) suggested background: MSU7.0 geneID (TIGR), (2) statistical test method: Fisher’s exact test, (3) multitest adjustment method: Yekutieli (FDR under dependency), (4) significance level: 0.05, (5) minimum number of mapping entries: 5 and (6) GO type: Plant GOSlim. Additional functional information, such as pathway annotation, was retrieved from UniProt^48^ and the Kyoto Encyclopedia of Genes and Genomes (KEGG)^49^.

## Supplementary information

Supplementary Figures.

Supplementary Table S1.

Supplementary Table S2.

Supplementary Table S3.

Supplementary Table S4.
