# Supplementary material for: SWATH-MS-based quantitative proteomics reveals a uniquely intricate defense response in Cnaphalocrocis medinalis-resistant rice
Source: Sci Rep. 2020 Aug 5;10:6597. doi: 10.1038/s41598-020-63470-1 (PMC7406494; doi:10.1038/s41598-020-63470-1)
Supplement: Supplementary file 1 — Supplementary Figures. [file 41598_2020_63470_MOESM1_ESM.pdf]

Supplementary Figures

SWATH-MS-based quantitative proteomics reveals a uniquely intricate defense response in *Cnaphalocrocis medinalis*-resistant rice

Boon Huat Cheah, Hou-Ho Lin, Han-Ju Chien, Chung-Ta Liao, Li-Yu D Liu, Chien-Chen Lai, Ya-Fen Lin*, Wen-Po Chuang*

*Address for correspondence: Department of Agronomy, National Taiwan University, Taipei 10617, Taiwan; yafenlin0725@ntu.edu.tw; wenpo@ntu.edu.tw

Supplementary Figure S1

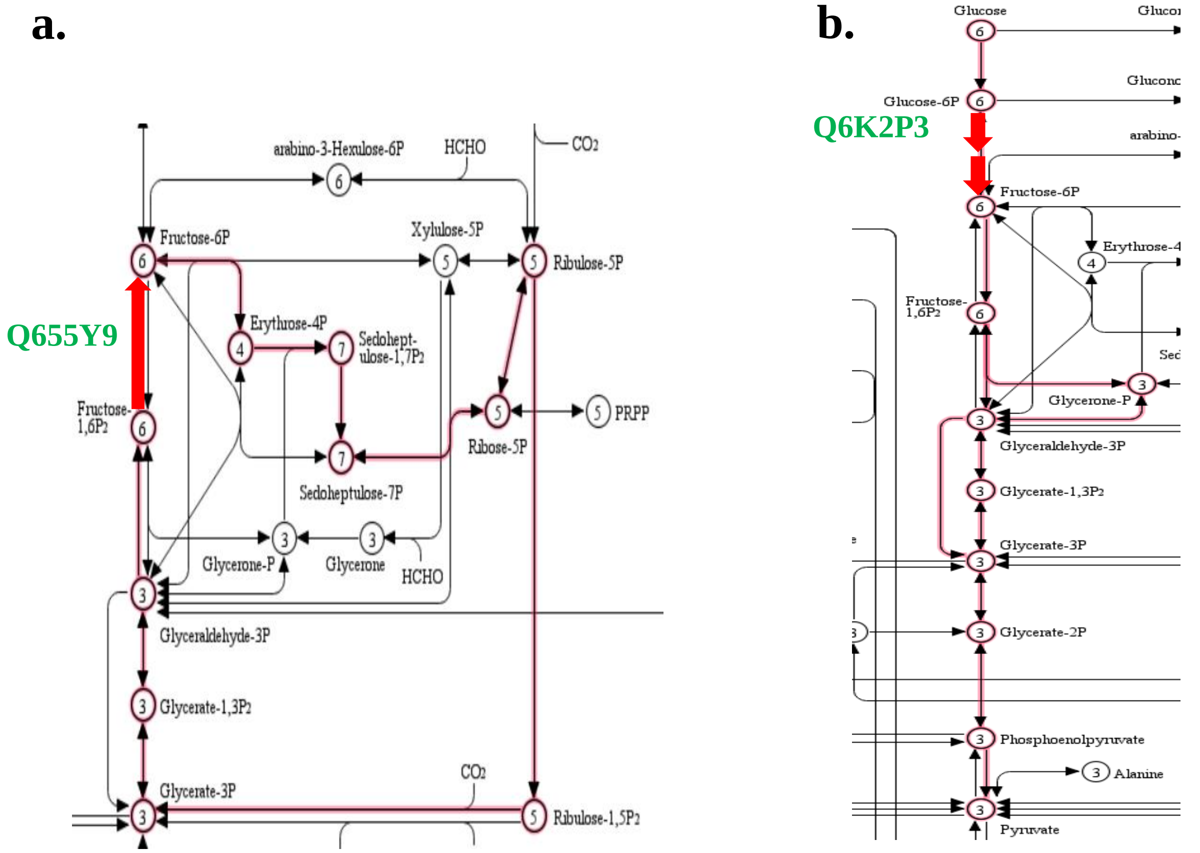

osa00710:
carbon fixation in photosynthetic organisms

osa00010:
glycolysis/gluconeogenesis

Supplementary Figure S1: KEGG pathway annotation of fructose 1,6-bisphosphatase (Q655Y9) and glucose-6-phosphate 1-epimerase (Q6K2P3) that were upregulated in Qingliu compared with TN1 at three time points of insect herbivory. (a) In carbon fixation process (osa00710), fructose 1,6-bisphosphatase (Q655Y9) converts fructose-1,6-bisphosphate to fructose 6-phosphate. **(b)** In glycolysis/gluconeogenesis (osa00010), Glucose-6-phosphate 1-epimerase (Q6K2P3) catalyses the isomerisation of alpha-D-glucose 6-phosphate to beta-D-glucose 6-phosphate which can then be converted to fructose 6-phosphate. Copyright permission was obtained for the KEGG pathway maps [49].

Supplementary Figure S2

a. GO biological process enrichment for upregulated proteins at 0 h

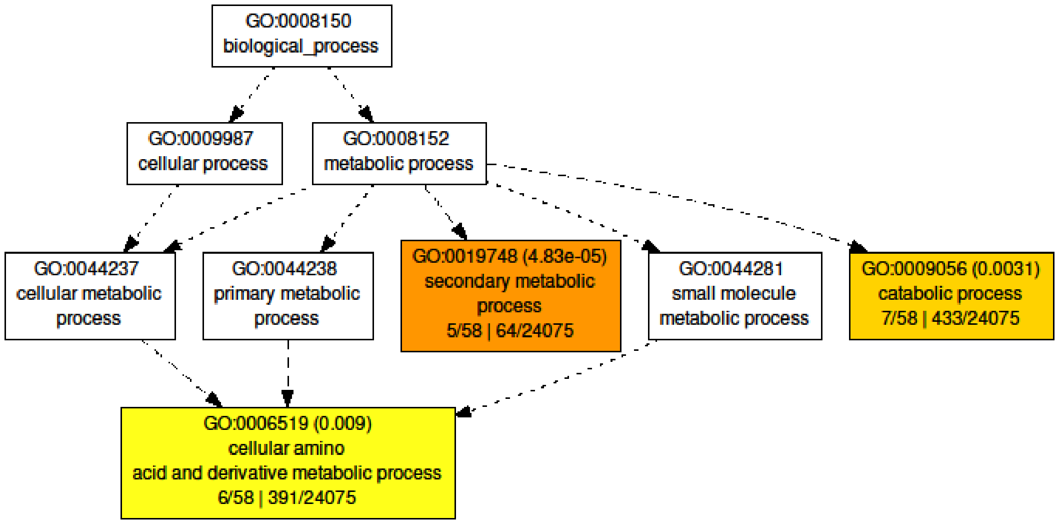

b. GO biological process enrichment for downregulated proteins at 0 h

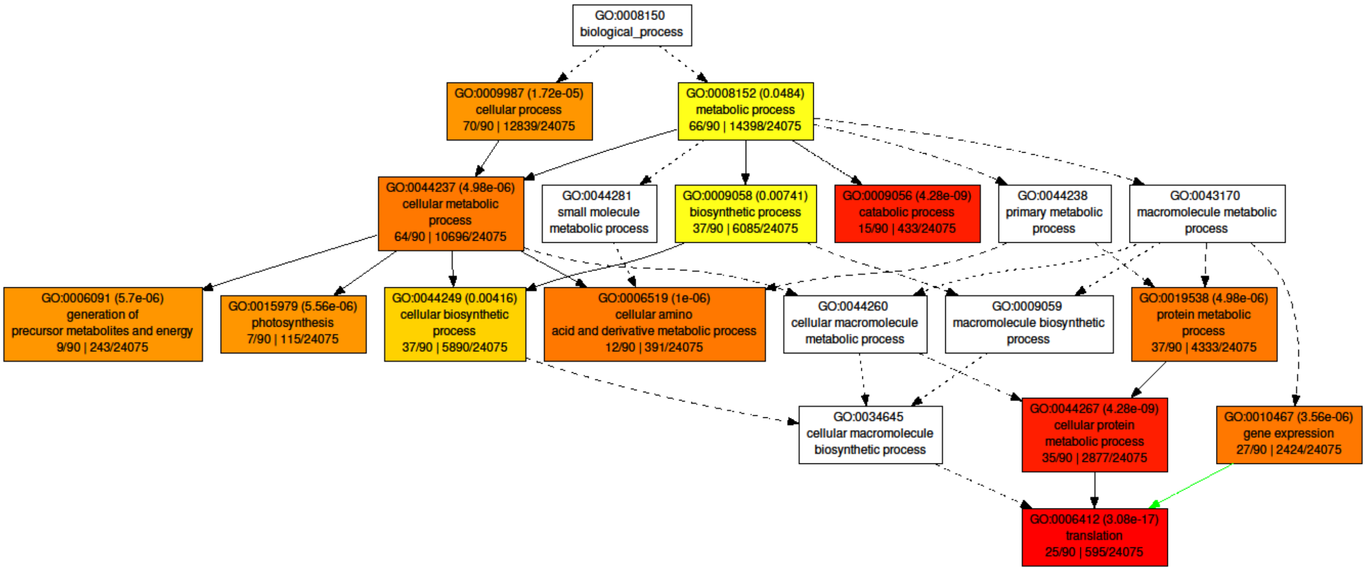

c. GO biological process enrichment for upregulated proteins at 6 h

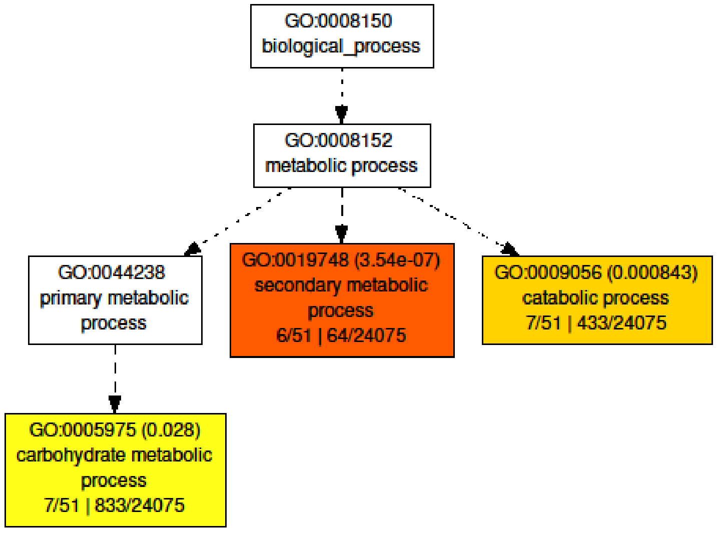

d. GO biological process enrichment for downregulated proteins at 6 h

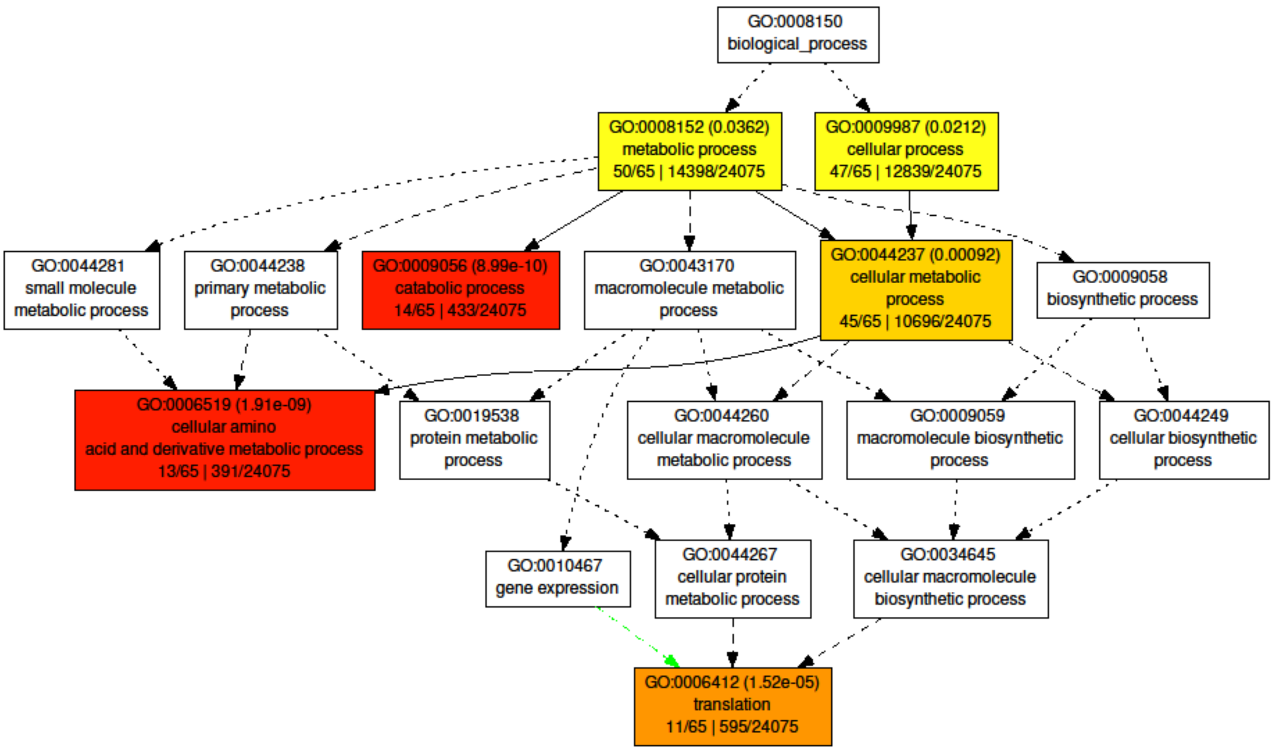

e. GO biological process enrichment for upregulated proteins at 24 h

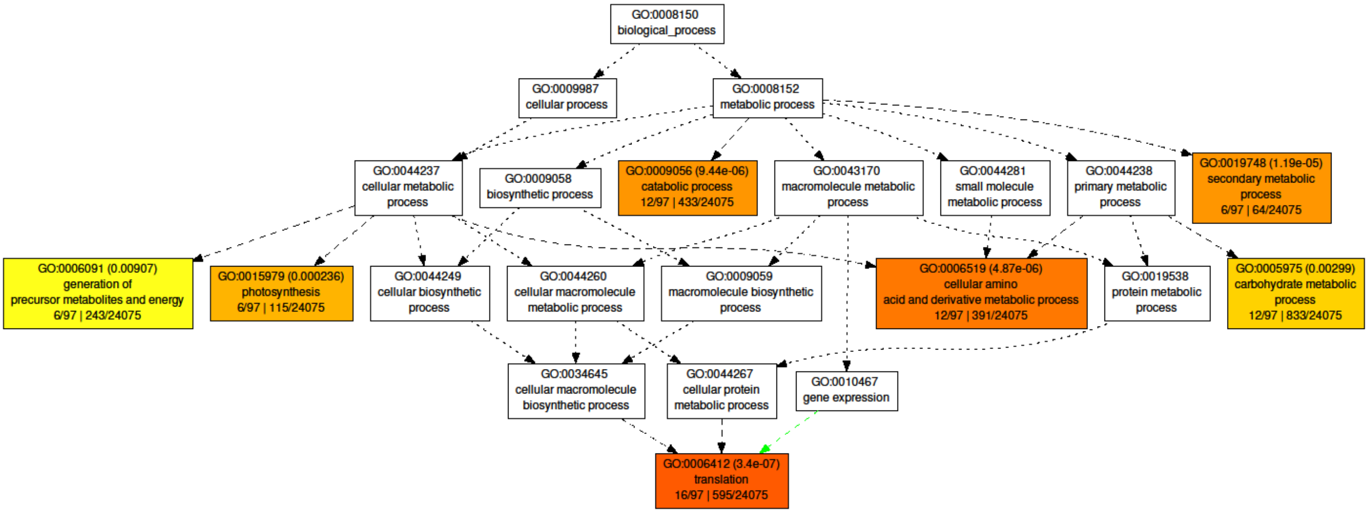

f. GO biological process enrichment for downregulated proteins at 24 h

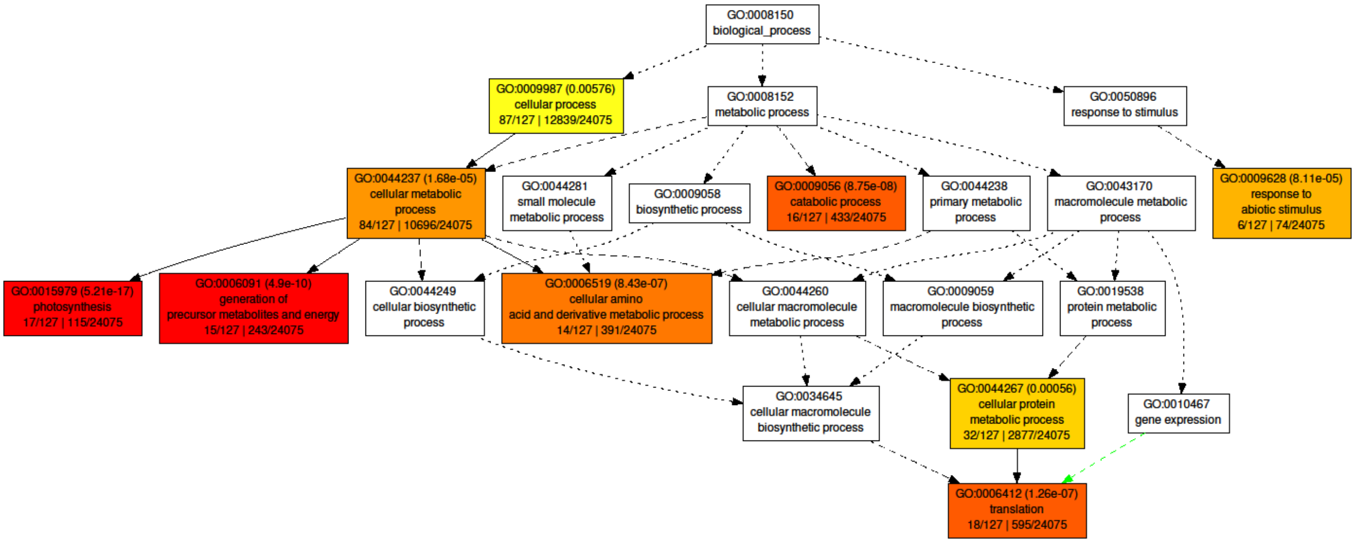

g. GO biological process enrichment for upregulated proteins at 72 h

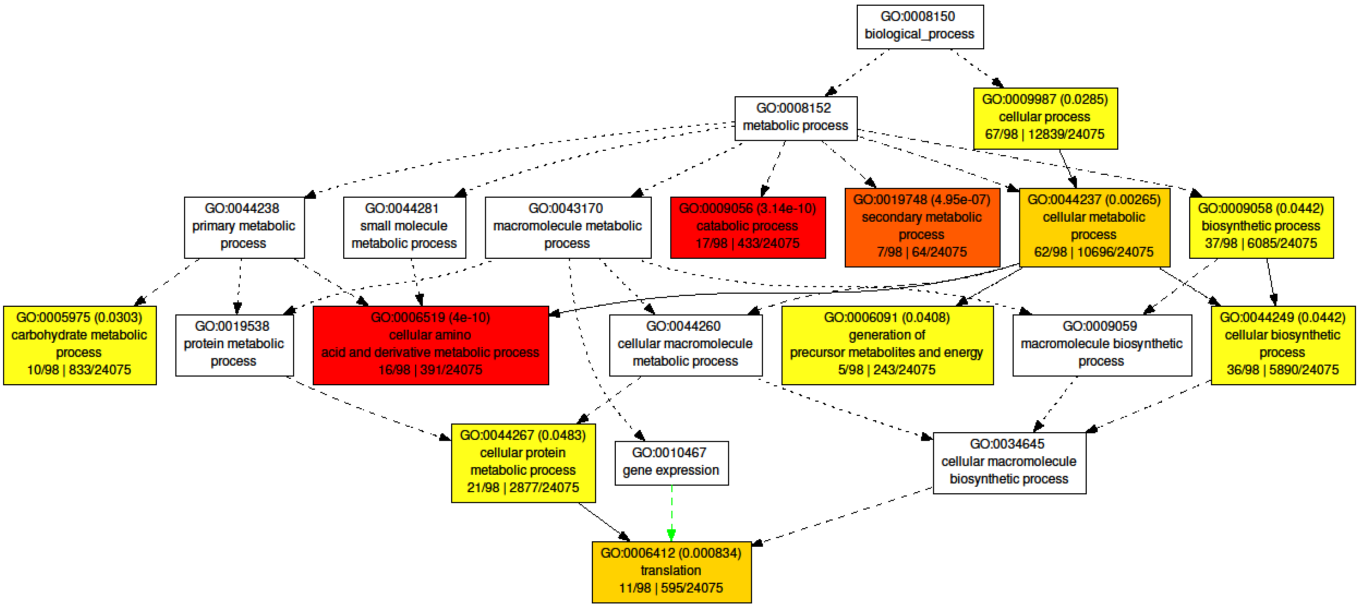

h. GO biological process enrichment for downregulated proteins at 72 h

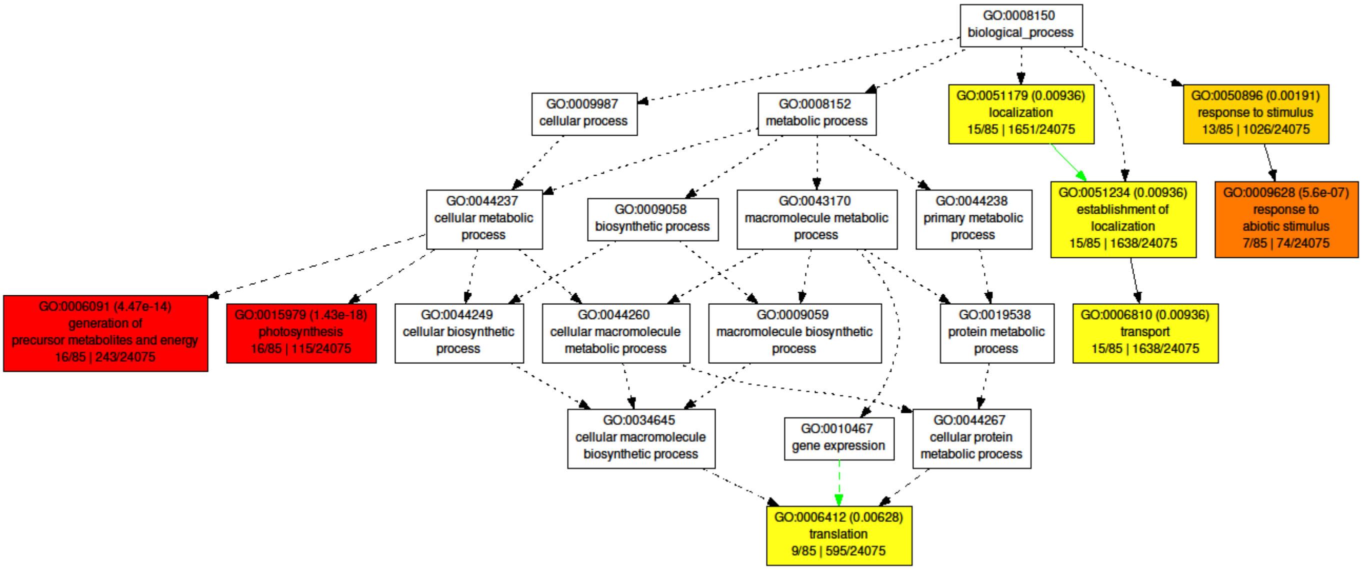

Supplementary Figure S2: Flowcharts of the enrichment of GO biological processes for upregulated and downregulated proteins in Qingliu compared with TN1 at each time point of insect herbivory. GO biological process enrichment **(a)** for upregulated proteins at 0 h, **(b)** for downregulated proteins at 0 h, **(c)** for upregulated proteins at 6 h, **(d)** for downregulated proteins at 6 h, **(e)** for upregulated proteins at 24 h, **(f)** for downregulated proteins at 24 h, **(g)** for upregulated proteins at 72 h and **(h)** for downregulated proteins at 72 h. The increasing significance of enrichment (adjusted p-value) is depicted in the order of yellow, orange and red boxes.
